# Monitoring of Women with Anti-Ro/SSA and Anti-La/SSB Antibodies in Germany—Status Quo and Intensified Monitoring Concepts

**DOI:** 10.3390/jcm13041142

**Published:** 2024-02-17

**Authors:** Ivonne Alexandra Bedei, David Kniess, Corinna Keil, Aline Wolter, Johanna Schenk, Ulrich J. Sachs, Roland Axt-Fliedner

**Affiliations:** 1 Department of Prenatal Diagnosis and Fetal Therapy, Justus-Liebig University Giessen, 35392 Giessen, Germany; aline.wolter@gyn.med.uni-giessen.de (A.W.); johanna.schenk@gyn.med.uni-giessen.de (J.S.); roland.axt-fliedner@gyn.med.uni-giessen.de (R.A.-F.); 2 Department of Prenatal Diagnosis and Fetal Therapy, Philipps-University Marburg, 35041 Marburg, Germany; kniessd@students.uni-marburg.de (D.K.);; 3 Institute for Clinical Immunology and Transfusion Medicine, Justus-Liebig University, 35392 Giessen, Germany; ulrich.sachs@immunologie.med.uni-giessen.de; 4 Center for Transfusion Medicine and Hemotherapy, University Hospital Giessen and Marburg, 35041 Marburg, Germany; 5 German Center for Fetomaternal Incompatibility (DZFI), University Hospital Giessen and Marburg, 35392 Giessen, Germany

**Keywords:** rheumatic disease in pregnancy, fetal atrioventricular block, home monitoring, anti-Ro/anti-La antibodies, lupus erythematosus, Sjögren syndrome

## Abstract

**Background:** The fetuses of pregnant women affected by anti-Ro/anti-La antibodies are at risk of developing complete atrioventricular heart block (CAVB) and other potentially life-threatening cardiac affections. CAVB can develop in less than 24 h. Treatment with anti-inflammatory drugs and immunoglobulins (IVIG) can restore the normal rhythm if applied in the transition period. Routine weekly echocardiography, as often recommended, will rarely detect emergent AVB. The surveillance of these pregnancies is controversial. Home-monitoring using a hand-held Doppler is a promising new approach. **Methods:** To obtain an overview of the current practice in Germany, we developed a web-based survey sent by the DEGUM (German Society of Ultrasound in Medicine) to ultrasound specialists. With the intention to evaluate practicability of home-monitoring, we instructed at-risk pregnant women to use a hand-held Doppler in the vulnerable period between 18 and 26 weeks at our university center. **Results:** There are trends but no clear consensus on surveillance, prophylaxis, and treatment of anti-Ro/La positive pregnant between specialists in Germany. Currently most experts do not offer home-monitoring but have a positive attitude towards its prospective use. Intensified fetal monitoring using a hand-held Doppler is feasible for pregnant women at risk and does not lead to frequent and unnecessary contact with the center. **Conclusion:** Evidence-based guidelines are needed to optimize the care of anti-Ro/La-positive pregnant women. Individual risk stratification could help pregnancy care of women at risk and is welcmed by most experts. Hand-held doppler monitoring is accepted by patients and prenatal medicine specialists as an option for intensified monitoring and can be included in an algorithm for surveillance.

## 1. Introduction

Around 1% of all women are positive for anti-Ro (anti-Sjögren’s syndrome-related antigen A/SSA) [1,2,3]. For women with a known rheumatic disease, the prevalence is around 40% for patients with systemic lupus erythematosus (SLE) and, in patients with Sjögren Syndrome, it may be as high as 100% [2,4]. Due to the FcRn-associated transplacental transport of anti-Ro (anti SSA) and anti-La (anti SSB) antibodies, fetuses are at risk of transient and permanent organ damage. The most severe manifestations are irreversible complete congenital heart block (CHB) and dilated cardiomyopathy. Around 1–2% of fetuses of antibody-positive primigravid mothers, or multipara without a previously affected off-spring, are affected [5,6]. Other cardiac manifestations can be valve dysfunction, sinus bradycardia and endocardial fibroelastosis (EFE) [7,8,9,10,11]. Conduction system and myocardial disease can lead to fetal or neonatal death and a lifelong need for cardiac pacing or even heart transplantation [11]. The mortality rate rises with diagnosis at a gestational age < 20 weeks, ventricular rate < 50 bpm, fetal hydrops and impaired left ventricular function [12]. The recurrence rate of fetal CHB in mothers with a previously affected fetus is around 16–18% [7,8,11,13]. Hydroxychloroqine (HCQ) reduces the recurrence of CHB in anti-SSA/Ro-exposed pregnancies by more than 50% and is recommended as a secondary prophylaxis [14]. Anti-Ro/anti-La antibodies start crossing the placenta late in the first trimester [15]. They bind to their corresponding antigens on cardiac cells that have undergone apoptosis, a process which translocates the otherwise intracellularly positioned Ro and La antigens to the cell surface. These immune complex-bearing cardiomyocytes are then phagocytosed by macrophages. Downstream signaling generates the production of pro-inflammatory and pro-fibrosing cytokines [11,16,17]. Another theory is that antibodies cross-react with L-type calcium channels (LTCC) [2,18]. The histological correlate of CHB is the antibody-mediated inflammation and fibrosis of the AV node [7,15,19]. Once a complete CHB has developed, the condition is irreversible and potentially no longer responsive to anti-inflammatory treatment. Incomplete CHB seems to be reversible [20,21]. More recently, FcRn-blocking agents have been discussed to offer an effective management strategy for immune-mediated heart block [22]. There appears to be a “window of opportunity” in the transition to complete CHB in which anti-inflammatory therapy (IVIG and fluorinated steroids) can consolidate the status quo, namely second degree CHB, or even restore sinus rhythm [21,23,24,25,26,27,28,29]. Even though there is a consensus that cardiac affections in neonatal lupus is a potentially life-threatening condition, there is no consensus on prenatal treatment [30].

It was previously assumed that the development of CHB was a slow-developing and measurable continuum from the prolongation of AV conduction time to complete CHB and the approach of surveillance by weekly echocardiography, measuring the AV conduction time, was based on this idea. In contrast to this assumption, it has been shown that fetal AV prolongation does not reliably predict progressive heart block [31,32]. It is unclear whether first-degree heart block detected in utero also progresses to more advanced heart block [2]. The transition time from incomplete CHB to irreversible CHB can occur in hours [21,27,29].

The surveillance of at-risk women is controversial and there is no clear consensus on how to monitor these pregnancies [33,34]. Intense surveillance by weekly echocardiography is frequently recommended between (16) 18–26 weeks GA [15,19,28,31,35]. Considering that complete CHB can develop in hours, the time interval for potentially successful intervention is short and will be missed by this approach. The event rate is only 1–2%, so weekly examinations will overtreat most at-risk pregnant women, which is not cost-effective and cannot successfully prevent the development of complete CHB.

To perform surveillance of at-risk pregnancies more individually and precisely, efforts have been made to find additional predictors of risk and guide the surveillance of these women based on their individual risk profile. The quantification of antibodies and definition of a specific risk cut-off seems to be a promising approach [20,36,37]. In order to detect emerging complete CHB in a timely manner, the more frequent control of the fetal heart rate using a hand-held Doppler seems to be a promising option [19,21].

In Germany, home monitoring using a hand-held Doppler is not yet offered as routine in monitoring these high-risk pregnancies. A universally applied guideline does not exist.

Our study has two objectives:

1. To determine the status quo for the surveillance, prevention, and treatment of pregnant women with anti-Ro/La antibodies and their fetuses by ultrasound specialists in Germany and their willingness to offer home monitoring in their patients.
2. To evaluate the requirements and practicability of home monitoring at a single university center in Germany.

## 2. Materials and Methods

Part 1: We conducted a quantitative cross-sectional survey-based study. Data were collected prospectively using an online questionnaire between 6 March 2023 and 31 March 2023.

The internet link was sent by the “German Society for Ultrasound in Medicine” (DEGUM) to its members by email. The survey was performed via the online platform “LamaPoll” and was addressed at ultrasound specialists with DEGUM level I–III who take care of pregnant women with anti-Ro/SSA and/or anti-La/SSB antibodies. DEGUM has different levels of expertise, with level I being the most basic and level III the most expert level. In Germany, it is common practice that the care of these women is not provided by pediatric cardiologists, but by specialists of prenatal diagnosis and fetal therapy.

Participation was completely anonymous. Persons without DEGUM level and those who do not manage anti-Ro/SSA and/or anti-La/SSB antibody-positive pregnant women were excluded.

The questionnaire contained a total of 19 questions (see Supplementary Material Section S1).

Part 2: To evaluate the feasibility of home monitoring using a hand-held Doppler, we initiated a prospective single-center study of anti-Ro/SSA and/or anti-La/SSB-positive pregnant women, which were recruited from January 2021 to December 2023. The patients were recruited from the department of prenatal diagnosis and fetal therapy of the University Hospital Giessen and Marburg (UKGM) in Germany. The study was approved by the ethics committee of Justus-Liebig University, Giessen, Germany, AZ 225/21 and Philipps University, Marburg, Germany, AZ 77/22.

Inclusion criteria:

- Pregnant women with singleton pregnancies and known rheumatic disease in whom anti-Ro/SSA and/or anti-La/SSB antibodies have been detected.
- Gestational age between 16 and 26 weeks.
- Age ≥ 18 years.

Exclusion criteria:

- Pregnant women with known rheumatic disease, without evidence of anti-Ro/SSA and/or anti La/SSB antibodies.
- Gestational age ≥ 26 weeks.
- Multi-fetal pregnancies.
- Fetal conduction system disease already present in the current pregnancy.
- Declined to sign the consent form.

Initial visit:

Patients with a known rheumatic disease and confirmed antibody positivity were offered study participation. After extensive counselling, patients were included if they had signed the study consent form.

Blood samples were analyzed for the presence of anti-SSA/SSB antibodies in a pooled approach (ENA pool) using a standardized ELISA method (Euroimmun, Lübeck, Germany). Samples with a result above the decision limit were examined for the specific presence of anti-Ro50/Ro62 antibodies using a standardized chemiluminescence method (Werfen, Barcelona, Spain).

A detailed medical history was taken, using an independently designed medical history form, which included questions on demographics, rheumatic disease, current medication and previous pregnancies (Supplementary Material Section S2).

At initial visit, detailed ultrasounds, including fetal echocardiography to evaluate the cardiac anatomy, rhythm and function, were obtained in all cases. Fetal echocardiography was performed by an expert in fetal medicine (R.A-F, I.B., A.W. and J.S.). Fetal heart rate was determined from measures of consecutive Doppler waveforms of the umbilical artery. The AV interval, defined as the time between the onsets of atrial and ventricular contraction, was measured from the onset of the mitral wave to the onset of the aortic outflow from the simultaneous mitral and aortic outflow Doppler wave forms.

To be sure that, at the moment of immediate need, the therapy will be covered by the insurance company, an application for potential cost coverage for off-label IVIG therapy was sent to the patient´s health insurance company at the moment of study inclusion.

The patients received extensive training on self-monitoring using a hand-held Doppler device at 16 weeks gestational age. In cases of a previously affected child, women were instructed to start home monitoring from the 16th week of pregnancy, while women who had not given birth to an affected child began home monitoring at 18 weeks. More specifically, the mothers were instructed to measure the fetal heart rate three times a day using a hand Doppler and give feedback. They were requested to document the fetal heart rate in a weekly documentation sheet, which was checked at each follow-up appointment at the study center.

On this sheet, patients could also indicate, via a scaled question, how they felt about home monitoring. The scale ranged between “I fully agree” to “I do not agree at all” (Supplementary Material Section S3).

To distinguish a regular rhythm from an irregular rhythm, the women were shown videos and audio recordings of normal and abnormal fetal heart rates. In cases where the fetal rhythm was abnormal, or the fetal heart rate was below 100 bpm, the patients were instructed to present at our center within 6 h to confirm or exclude abnormal heart rhythm and receive anti-inflammatory treatment within twelve hours.

The documentation forms contained a table for documenting the measurements for a period of one week, as well as questions regarding whether the women felt strengthened or stressed by the measurements. At each subsequent visit, mothers reviewed the results of the previous week(s) of monitoring. Any contact with the center was documented.

After 26 weeks of gestation, home monitoring by the mothers was completed. Thereafter, controls at our center were scheduled in accordance with the disease status, but normally every 4 weeks.

**Figure 1:**
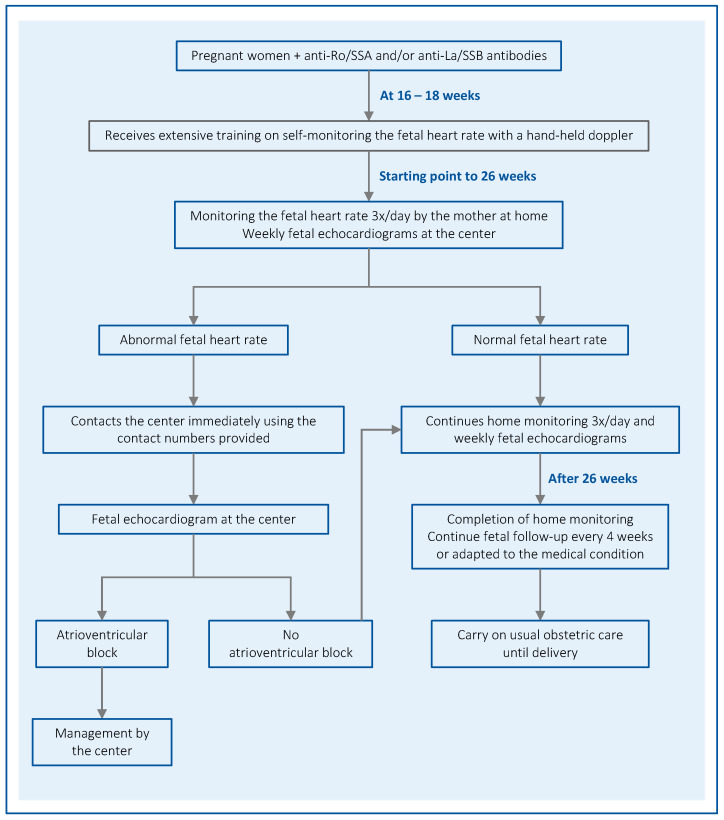
Algorithm for monitoring pregnant women with anti-Ro/anti-La antibodies.

After birth, the infants were seen by our neonatologists and received a cardiac evaluation, including an electrocardiogram, performed by a pediatric cardiologist (Figure 1).

All data were de-identified and entered into a local database. Statistical analysis was performed using Excel spreadsheet mathematic functions (Version 16.69.1). Descriptive statistics were calculated using mean values, SD and minimum and maximum for continuous variables and N (%) for categorical variables.

## 3. Results

### 3.1. Web-Based Survey

#### 3.1.1. Setting and Qualification

A total of 114 ultrasound specialists with DEGUM level I–III took part in our online survey; 6 out of 113 (5.3%) participants had DEGUM Level I, 99 (87.6%) had DEGUM Level II and 7 (6.2%) had Level III. One (0.9%) participant did not give any details and was excluded from analysis. Of the 113 specialists, 64 (56.6%) work in an outpatient setting; the remaining 49 (43.4%) work in a hospital setting. Of the 111 participants, 64 (57.7%) work with a laboratory that gives a quantitative or semiquantitative analysis of anti-Ro/La antibodies. A total of 10 (9%) just gave positive or negative results without further quantification and 37 (33.3%) stated that they did not know. Of the 113 participants, 91 (80.5%) treat one to five at-risk women per year; 16 (14%) treat six or more.

#### 3.1.2. Prevention and Treatment

**Figure 2:**
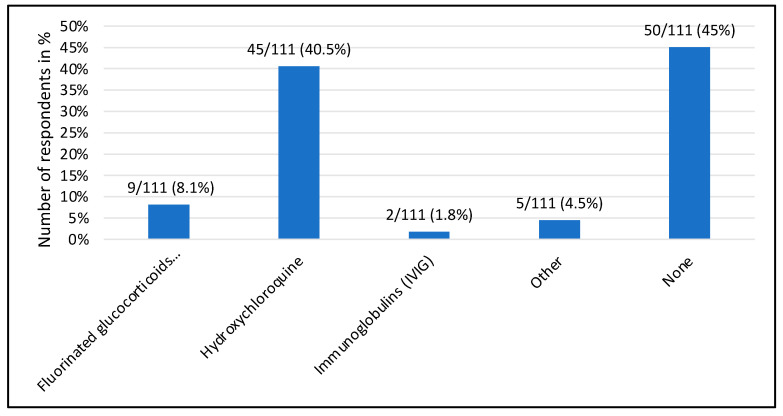
Drug prophylaxis considered appropriate for an anti-Ro/La positive primigravida or a multiparous pregnant woman without affected offspring.

Of the 111 specialists, 45 (40.5%) consider prophylaxis with hydroxychloroquine to be useful in the case of a seropositive primigravida, while 50 (45%) do not recommend any drug prophylaxis and 9 (8%) would start fluorinated steroids as prophylaxis. (Figure 2).

**Figure 3:**
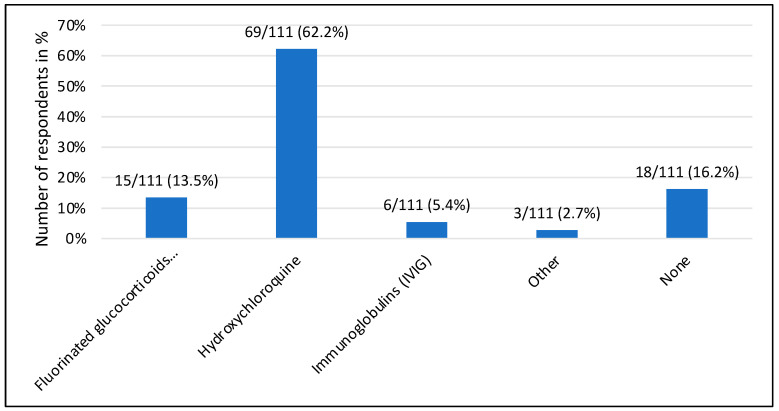
Drug prophylaxis considered appropriate for anti-Ro/La-positive pregnant women with affected offspring.

In the case of a mother who has had a previous affected pregnancy, 69 of 111 (62.2%) experts would support preventive treatment with hydroxychloroquine, 15 (13.5%) would give fluorinated steroids and 18 (16%) would not give any prophylaxis at all (Figure 3).

The data on the treatment of congenital AV block I–III° are summarized in Table 1.

When asked how quickly a complete AV-block can develop, 18 of 110 (16.4%) participants answered with less than 12 hours, 42 (38.2%) with 12 to 24 h, 10 (9.1%) with over 24 h and 40 (36.4%) stated that they did not know. However, 67 of 110 (61%) believe that the course of the disease and the prognosis of the fetus is more favorable with early treatment.

#### 3.1.3. Pregnancy Monitoring

Out of the 110 participating specialists, 52 (47.3%) perform weekly echocardiography in anti-Ro/anti-La antibody-positive pregnant women, 40 (36.4%) prefer controls every other week, 10 (9.1%) follow a different schedule, 3 (2.7%) do not perform any intensified monitoring and 5 (4.6%) do not think intensified monitoring is useful. The current practice of weekly fetal echocardiography with monitoring of the AV conduction is considered beneficial by 50 of 95 (52.6%) specialists, but not by 18 (19%). The remainder did not provide any information. Of the 110 participants, 103 (93.6%) do not instruct at-risk pregnant women to undergo home monitoring using a handheld Doppler. Only seven (6.4%) stated that they do so. However, 73 of 109 (67%) respondents could imagine instructing affected patients in self-monitoring in the future. Some participants had concerns about implementing home monitoring; these are summarized in Table 2.

#### 3.1.4. Immunoglobulin (IVIG) Therapy, Antibody Quantification and Risk Cut-off

When asked whether the participants could start treatment with immunoglobulins within twelve hours in the setting/infrastructure in which they work, 60 of 109 (55%) respondents answered that they could provide IVIG within twelve hours and 49 (45%) answered that they could not. Of the 109 participants, 98 (89.9%) are in favor of a cut-off for anti-Ro/anti-La antibodies for a specific risk classification and risk-adapted monitoring.

#### 3.1.5. Suggestions and Comments

Of the 96 participating ultrasound specialists, 25 (26%) left specific suggestions or comments on the subject. Of those, six (24%) reported that they are interested in the results of the survey and would like to be informed about it. Another six (24%) would like to have standardized algorithms or guidelines for the care of pregnant women with ant-Ro/anti-La antibodies.

### 3.2. Home Monitoring

#### 3.2.1. Recruitment and Participation

From January 2021 to December 2023, 15 women with positive anti-Ro/La antibodies presented at our center. Twelve study participants were recruited and monitored by our study center. Three pregnant women declined study participation. One woman declined to take part in the study because she would be moving to another federal state during the study period and the distance to our center would be too far. Another patient moved abroad during the monitoring phase. The third patient did not give any reasons for her refusal. Of those recruited, eleven (91.7%) completed the study. One patient did not complete the home monitoring without giving a reason.

#### 3.2.2. Characteristics of the Study Group

The maternal characteristics are summarized in Table 3.

Most mothers were of Caucasian origin (nine out of twelve; 75%) and fluently spoke German (ten out of twelve; 83.3%). The average maternal age at the beginning of the study was 32.2 ± 4 years (range between 23 and 39 years). The mean gestational age at the initial visit of the patient was 16 ± 4.1 weeks of gestation and, at the beginning of home monitoring, 18.3 ± 2.9 weeks of gestation. Six out of twelve (50%) of the enrolled women were primigravida. The most common rheumatic disease among the mothers was lupus erythematosus in eight women (66.7%). Treatment was carried out by the rheumatologist and already established when patients presented to our clinic. In two of the twelve (16.7%) pregnant women, treatment with hydroxychloroquine was initiated in order to prevent evolving CAVB from the rheumatologist, even though there was no affected previous pregnancy. Four out of twelve (33.3%) mothers received hydroxychloroquine already before the current pregnancy as part of their disease-related treatment regimen. Three out of twelve (25%) were treated with methylprednisolone. None of the enrolled women had a previously affected child. Four mothers had a previous miscarriage. Nine out of twelve (75%) women had planned their current pregnancy. Eight women (66.7%) reported that they had a consultation with their gynecologist or rheumatologist before their pregnancy; four (33.3%) did not. When questioned whether the mothers felt sufficiently informed by their gynecologist about special aspects of the course of the pregnancy or pregnancy monitoring, eight (66.7%) women felt well-informed while four (33.3%) did not. Regarding the question of whether the women felt adequately educated by their rheumatologist, nine (75%) women confirmed that they did.

#### 3.2.3. Results of Home Monitoring

**Figure 4:**
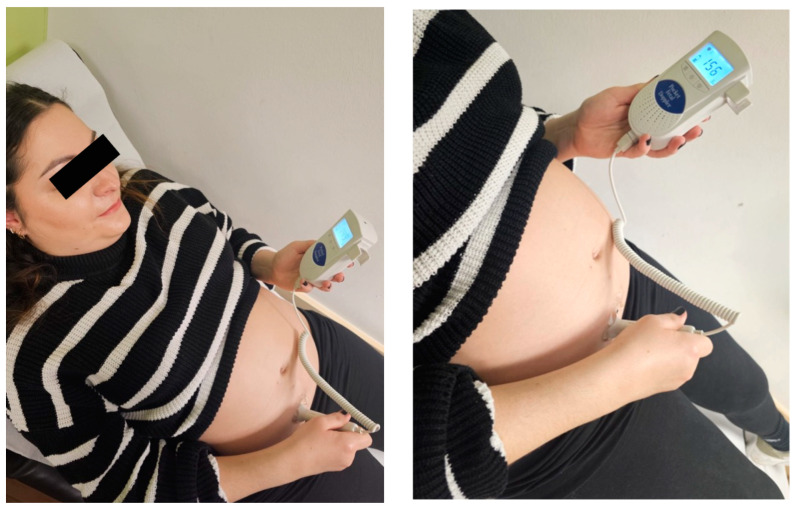
Patient measuring fetal heart rate using handheld Doppler.

The twelve enrolled mothers returned a total of 83 weekly documentation forms. Of these women, 82% fully completed the home monitoring protocol—measuring the fetal heart rate three times a day using a handheld Doppler. This means that they carried out 1428 of a total of 1743 fetal heart rate measurements. Figure 4 shows a patient using the handheld doppler device. The reasons for not performing fetal heart rate measurements included that the women forgot the Doppler device while travelling. For multiparous women, their daily routine with another infant was the main reason for omitting measurements. None of the patients contacted the center during the entire period due to an abnormal fetal heart rate or problems/insecurities with measurement.

In 44 of 83 (53%) of the weekly documentation forms, the women agreed or fully agreed that they felt empowered by home monitoring. Additionally, 38 (46%) neither agreed nor disagreed and 1 (1.2%) did not feel empowered. In 12 of the 83 (14.5%) weekly responses, the mothers stated that they felt more stressed because of self-monitoring. In 25 (30.1%) of these responses, participants neither agreed nor disagreed, in 42 (50.6%) they disagreed and in 4 (4.8%) they strongly disagreed.

## 4. Discussion

Our study focused on the standard of care for pregnant women with anti-Ro/La antibodies in Germany and the feasibility of home monitoring in a university setting. Home monitoring in this context not only means fetal heart rate measurements by the pregnant woman herself several times a day, but also creating an environment in which the pregnant woman is sufficiently trained, can reach an institution 24 h a day and receives the appropriate control and medication in the presumed time slot. We also wanted to evaluate whether home monitoring leads to increased and possibly unnecessary contact (“false alarms”) with the medical facility. Given the extremely rare event rate and the small number of cases in our cohort, it was not the aim of this study to determine the success rate of the timely treatment of emergent CHB with anti-inflammatory medication.

There are several important findings in this survey and prospective surveillance study of anti-Ro/anti-La-positive pregnancies.

1. Regarding fetal surveillance, there were some trends but no clear consensus on how to monitor these pregnancies.

Approximately 50% of participating experts offer fetal echocardiography weekly, and 36% every other week. Furthermore, 9% have an individualized scheme and almost 3% do not offer intensified surveillance in the time between 18 and 26 weeks GA. Of the experts, 28% were not sure if the weekly control of AV conduction time is beneficial. Actually, home monitoring is offered by only 6% of them, but 67% have a positive attitude towards home monitoring and could imagine its prospective use in the future.

2. Regarding the medical prophylaxis of CHB, there was no consensus between the different experts. In a pregnant woman without affected off-spring (primigravida or non-affected previous pregnancy), 45% would offer no prophylactic treatment, 40% would offer hydroxychloroquine, 8% would offer fluorinated steroids and 2% would offer IVIG. In cases of an affected previous pregnancy, 62% would offer hydroxychloroquine and 16% would offer no prophylactic treatment.

3. Regarding the treatment of CHB, 58% would not treat a first-degree CHB, 38% would not treat a second-degree or emergent CHB and 51% would not treat a complete fetal heart block. In 61%, there was the belief that early detection and treatment would have a positive impact on overall prognosis. Of the respondents, 55% reported working in a setting where IVIG is available and can be administered within twelve hours.

4. Home monitoring using a hand-held Doppler three times a day could be carried out by most pregnant women, regardless of their origin or knowledge of the German language. Most participants had a positive or neutral attitude towards home monitoring. There was no increased rate of unnecessary contact/false positives with our center. The training of the patients could be easily integrated into our outpatient clinic setting.

Previously, the surveillance of anti-Ro/anti-La-positive pregnancies was founded on the hypotheses that anti-Ro/SSA-mediated CHB progresses over time and that treatment in the early stages could restore normal conduction. The PRIDE study showed that complete fetal CHB could develop in between the interval of weekly echocardiography and that there was no continuous transition from normal rhythm to prolonged AV conduction time to first-, second- and third-degree heart block [31]. Cuneo et al. could show that other cardiac affections associated with anti-Ro/La antibodies did not precede CHB but were detectable in fetuses with second- and third-degree CHB [21]. The definition of first-degree CHB is controversial [21,33]. While incomplete CHB is a potentially reversable condition, complete CHB is irreversible and not amenable to anti-inflammatory treatment [21,23,24,25,26,27]. The transition from normal rhythm to complete heart block can develop in less than twelve hours [21,27,29]. Cardiac fetal and neonatal lupus is exceedingly rare and affects only 1–2% of primigravid women or women with an unaffected previous offspring [5,6]. Weekly or bi-weekly fetal echocardiography, therefore, will only serendipitously detect emergent CHB. Most women under intense surveillance will never develop a problem and the ones in need would not be detected by this approach. This current practice, therefore, consumes both human and economic resources without any demonstrable benefit [38]. Treatment with fluorinated steroids not only has benefits, but also well-known side effects [24,39,40,41,42]. The dosage is largely determined empirically. Their use should be limited to a very well-defined group of patients in a research protocol. IVIG is expensive and not easily available in an outpatient setting. Even though it is mostly well tolerated, and most side effects like flushing, headache, chills and fever are transient, some rare serious side effects have been described [43]. Treatment with IVIG has been beneficial in a variety of immune-mediated diseases. Its presumed mechanism of action is based on the saturation of FcRn receptors and decreased fetal exposure to anti-SSA/Ro and anti-SSB/La by limiting placental transfer, increasing catabolism in the maternal circulation and attenuating the anti-inflammatory response [13,44]. In a low-dose regimen, it does not prevent the recurrence of CAVB [13]. Not all study protocols include IVIG in addition to steroids for the treatment of CHB, and there is no solid evidence regarding the dosage and duration of IVIG treatment [45]. As for steroids, these should be used within a research protocol. The only proven secondary prevention is starting with hydroxychloroquine before 10 weeks GA as a secondary prophylaxis in women with a previously affected fetus [14].

Costedoat-Chalumeau et al., therefore, questioned the dogma of current surveillance practice [33]. They advocate for an individualized approach in a scientific setting and consider home monitoring to be a promising approach. Evers et al. showed that current screening strategies are not efficient nor cost-effective, and they support risk stratification by antibody quantification [38]. Clowse et al. and Carvalho et al. could show trends but no consensus regarding the surveillance, prevention and treatment of women with anti-Ro/La antibodies [34,46]. This is in accordance with the findings of our survey. Cuneo et al. could show that the ambulatory fetal heart rate surveillance of anti-SSA-positive pregnancies is feasible, has a low false positive rate and is empowering to mothers [19]. In our study on the feasibility of home monitoring in a single-center university setting, we have been able to confirm this observation.

### Strengths and Limitations

This is the first survey evaluating the surveillance of women with anti-Ro/La antibodies in Germany. It gives insight into the current practice and illuminates differences in the treatment approaches of a high number of highly qualified participants working in different clinical settings. Based on these findings, we can show that there is an imminent need for a guidelines, the homogenization of surveillance and care and treatment based on individual risk and not solely on the single fact that a woman is anti-Ro/La-positive. This includes the quantification of antibodies, which we have recently implemented, and home monitoring using a hand-held Doppler with the possibility of being seen and treated within 12 h after an abnormal heartbeat is detected.

A limitation is the small number of participants in our single-center clinical prospective trial. Our university setting with 24 h service, and the availability of IVIG whenever needed, may not be applicable to other settings. Our findings must be confirmed in a study with a larger number of patients seen in different clinical contexts.

## 5. Conclusions

There are some trends, but there is no clear consensus, with respect to the surveillance, prophylaxis and treatment of pregnant women with anti-Ro/La antibodies, between specialists in prenatal ultrasound in Germany. Intensified fetal monitoring using a hand-held Doppler is feasible and does not lead to frequent and unnecessary contact with the center. Most women could carry out measurements without an important negative impact on their daily life. This approach is accepted by patients and prenatal medicine specialists as an option for intensified monitoring and can be included in an algorithm for surveillance. Individual risk stratification, for example, through antibody quantification, can help to individualize patients’ surveillance and is welcomed by most experts. Evidence-based guidelines are needed to optimize the care of anti-Ro/La-positive pregnant women.

## Figures and Tables

**Table 1:** Treatment of congenital AV block I–III°.

AV block I°	*n* = 109 (%)
No treatment	63 (57.8)
Fluorinated glucocorticoids	27 (24.8)
IVIG	3 (2.8)
Fluorinated glucocorticoids + IVIG	9 (8.3)
Hydroxychloroquine	7 (6.4)
Betamimetics	0 (0)
**AV block II°**	***n* = 108 (%)**
No treatment	41 (38)
Fluorinated glucocorticoids	40 (37)
IVIG	5 (4.6)
Fluorinated glucocorticoids + IVIG	22 (20.4)
Betamimetics	0 (0)
**AV block III°**	***n* = 108 (%)**
No treatment	55 (50.9)
Fluorinated glucocorticoids	22 (20.4)
IVIG	4 (3.7)
Fluorinated glucocorticoids + IVIG	24 (22.2)
Betamimetics	3 (2.8)

Abbreviations: AV block, Atrioventricular block; IVIG, Immunoglobulins.

**Table 2:** Concerns regarding home monitoring.

	*n* = 42 (%)
No consequences	9 (21.43)
Measurement uncertainties/many false positive findings	15 (35.71)
Increases anxiety of patients	9 (21.43)
No convincing evidence so far	7 (16.67)
Too time-consuming to implement	2 (4.76)

**Table 3:** Maternal Characteristics (*n* = 12).

	Mean, SD, (Min-Max) or *n* (%)
**Maternal age (years)**	32.2 ± 4 (23–39)
**BMI**	26 ± 4.4 (19.8–33.7)
**Gestational age at initial visit (weeks)**	16 ± 4.1 (10–23)
**Gestational age at beginning of home monitoring (weeks)**	18.3 ± 2.9 (11–22)
**Race**	
Caucasian	9 (75)
Asian	2 (16.7)
More than 1 race	1 (8.3)
**Highest school degree**	
High school	5 (41.7)
University degree	7 (58.3)
**Distance to the study center**	
<50 km	9 (75)
50–100 km	2 (16.7)
>100 km	1 (8.3)
**German language**	
Poor knowledge	1 (8.3)
Moderate knowledge	1 (8.3)
Fluent	10 (83.3)
**Diagnosis**	
Lupus erythematosus	8 (66.7)
Antiphospholipid syndrome	1 (8.3)
Sjögren syndrome	2 (16.7)
Rheumatoid arthritis	1 (8.3)
**Obstetrical history**	
Primigravida	6 (50)
Multigravida	6 (50)
**Affected previous pregnancy**	0 (0)
**Previous miscarriages**	4 (33.3)

Abbreviations: BMI, body mass index.

## Data Availability

The data used to support the findings in this study are available from the corresponding author upon reasonable request.

## Supplementary Materials

The following supporting information can be downloaded at https://www.mdpi.com/article/10.3390/jcm13041142/s1, Section S1: online survey for DEGUM specialists, Section S2: Questionnaire for study participants, Section S3: Weekly documentation form.
