# Supplementary material for: Monitoring of Women with Anti-Ro/SSA and Anti-La/SSB Antibodies in Germany—Status Quo and Intensified Monitoring Concepts
Source: J Clin Med. 2024 Feb 17;13(4):1142. doi: 10.3390/jcm13041142 (PMC10889801; doi:10.3390/jcm13041142)
Supplement: Supplementary file 1 [file jcm-13-01142-s001.zip › jcm-2828532-supplementary.pdf]

Supplemental Material Section S1: Online survey for DEGUM specialists

Question 1: Where do you work?

- Outpatient clinic
- Hospital

Question 2: Which DEGUM level do you have?

- DEGUM-Stufe I
- DEGUM-Stufe II
- DEGUM-Stufe III
- None

Question 3: How many pregnant women with anti-Ro/La antibodies do you see each year?

- None
- 1-5
- 6-10
- Over 10

Question 4: Does the laboratory you work with perform purely qualitative or also (semi-) quantitative measurements of antibodies (anti-Ro/anti-La)?

- Yes
- No
- I don´t know

Question 5 - Which drug prophylaxis do you consider appropriate for a primigravida with anti-Ro/anti-La antibodies?

- Fluorinated glucocorticoids (dexamethasone/betamethasone)
- Hydroxychloroquine
- Immunoglobulins (IVIG)
- Other: ______________________________
- None

Question 6 - What prophylactic medication do you consider useful for pregnant women with an affected previous pregnancy?

- Fluorinated glucocorticoids (dexamethasone/betamethasone)
- Hydroxychloroquine
- Immunoglobulins (IVIG)
- Other: ______________________________
- None

Question 7 - How often do you perform echocardiography on anti-Ro/La positive pregnant women between the (16th) 18th and 26th week of pregnancy?

- Weekly
- Every 2 weeks
- Different scheme

- I do not perform intensified monitoring
- I do not consider intensified monitoring by means of echocardiography to be useful

Question 8 - Do you instruct pregnant women on home monitoring (self-monitoring by a hand-held doppler device)?

- Yes
- No

Question 9 - Is this something you could imagine doing in the future?

- Yes
- No

→ If not, why not?

____

Question 11 - How do you treat an AV block I°?

- Not at all
- Fluorinated glucocorticoids (dexamethasone/betamethasone)
- Immunoglobulins (IVIG)
- Fluorinated glucocorticoids (dexamethasone/betamethasone) + IVIG
- Hydroxychloroquine
- Betamimetics

Question 12 - How do you treat an AV block II°?

- Not at all
- Fluorinated glucocorticoids (dexamethasone/betamethasone)
- Immunoglobulins (IVIG)
- Fluorinated glucocorticoids (dexamethasone/betamethasone) + IVIG
- Hydroxychloroquine
- Betamimetics

Question 13 - How do you treat a complete AV block?

- Not at all
- Fluorinated glucocorticoids (dexamethasone/betamethasone)
- Immunoglobulins (IVIG)
- Fluorinated glucocorticoids (dexamethasone/betamethasone) + IVIG
- Betamimetics

Question 14 – In your opinion, how quickly How quickly can a complete AV block develop?

- Under 12h
- 12-24h
- Over 24h
- I don´t know

Question 15 - Do you think that the course of the disease and the associated prognosis of the fetus can be improved by prompt diagnosis and treatment?

- Yes

- No
- I don´t know

Question 16 - Do you think the current practice of weekly fetal echocardiography with monitoring of PR conduction makes sense?

- Yes
- No
- I don´t know

→ If not, why not?

____

Question 17 – Are you able to start a treatment with immunoglobulins within 12 hours in the setting in which you work/the infrastructure in which you work?

- Yes
- No

Question 18 – Would you like to have a cut- off value for anti-Ro/anti-La antibodies for specific risk classification and risk-adapted monitoring?

- Yes
- No
- I don´t know

Question 19 - Suggestions and proposals.

Supplement Material Section S2: questionnaire for study participants

---

1. Age: _______
2. Height:_________
3. Weight:__________
4. Gestational age at initial visit:______________________________
5. Gestational age at the beginning of the study:__________________
6. Distance to the center: ○ up to 50km ○ 50-100km ○ over 100km
7. Ethnicity: ○ PoC ○ Hispanic ○ Caucasian ○ Asian ○

Mixed ○ Other:________________________

8. Highest level of education: ○ Secondary school ○ High school
○ University degree

9. Language skills

Language	No skills	Bad skills	Moderate skills	Good skills	Fluent
German	○	○	○	○	○
English	○	○	○	○	○
Turkish	○	○	○	○	○
Arabic	○	○	○	○	○
Other:	○	○	○	○	○
Other:	○	○	○	○	○

10. Medications:____

Was your medication changed due to the pregnancy? ○Yes ○No

If so, what has been changed:

____

____

____

11. Have you been diagnosed with a rheumatic disease? ○ Yes ○ No

If so, which:

a) Lupus erythematosus ○
b) Sjögren syndrome ○
c) Antiphospholipid syndrome ○
d) Rheumatoid arthritis ○
e) Other ○ : ____________________________

12. How long ago has your rheumatic disease been diagnosed? Since ____________________

13. Who has made the diagnosis? ____

14. Any previous pregnancies? ○ Yes ○ No

If yes:

Type of delivery: ○ Natural birth ○ C-section

Weight of the newborn:________________________

Gestational age at delivery:________________________________

Do you have a child affected by neonatal lupus? ○ Yes ○ No

Complications during pregnancy? ○ Yes
○ No

If yes:

○ Pre-eclampsia ○ Growth retardation ○ Hypertension

Other:____

15. Have you ever had a miscarriage before?? ○ Yes ○ No

If yes, at what gestational age?_________________

16. How many times have you been pregnant (before the current pregnancy)?

0 ○ 1 ○ 2 ○ 3 ○ 4 ○ 5 ○ more than 5 ○

17. How many births have you had?

0 ○ 1 ○ 2 ○ 3 ○ 4 ○ 5 ○ more than 5 ○

18. Have you and your partner ever had an ICSI (intracytoplasmic sperm injection)?

○ Yes. ○ No

19. Have you planned the current pregnancy?

○ Yes ○ No

20. Did your gynecologist/rheumatologist have a consultation with you before your pregnancy?

○ Yes ○ No

21. Do you think that your gynecologist has provided you with sufficient information about special aspects of the course of your pregnancy or monitoring?

○ Yes ○ No

22. Do you think that your rheumatologist has provided you with sufficient information about special aspects of the pregnancy process or monitoring?

○ Yes ○ No

23. Do you feel restricted in your everyday life by your illness?

○ Yes ○ No

24. Do you feel that your illness is a burden?

○ Yes ○ No

25. Do you worry a lot during this pregnancy?

Yes, I am worried a lot. ○	Yes, I am worried. ○	I am partly concerned, partly not. ○	No, I am not worried. ○	No, I am not worried at all. ○

Weekly documentation form

1. Please enter the date and the measured heart rates in the following table.

	Date:	Date:	Date:	Date:	Date:	Date:	Date:
**Heart rate**							
Morning							
Noon							
Evening							

2. How often were you not able to measure?

This week it was not possible for me to measure ______ times.

3. This week I have felt ______ by the measurement. (Please check the box)

	Totally agree	Agree	Neither agree nor disagree	Disagree	Don't agree at all
Stressed	O	O	O	O	O
Strengthened	O	O	O	O	O

4. Have you contacted the center?

	Yes	No
Contact	O	O
If so, how often?		

Evaluation of the documentation form

Non-subjective parameters:

1. Number of contacts made: ____

Why: ◯ Slow heart rate ◯ Irregular heart rate ◯ Heart rate not derivable

◯ Other: ________________________

2. Correct contacts:____________________
3. False alarm: ◯ Yes ◯No
4. If so, what reasons?
5. Were there any pregnant women who discontinued self-monitoring?

   ◯Yes ◯No

   If so, why? _____________________
